# Gene response profiles for *Daphnia pulex *exposed to the environmental stressor cadmium reveals novel crustacean metallothioneins

**DOI:** 10.1186/1471-2164-8-477

**Published:** 2007-12-21

**Authors:** Joseph R Shaw, John K Colbourne, Jennifer C Davey, Stephen P Glaholt, Thomas H Hampton, Celia Y Chen, Carol L Folt, Joshua W Hamilton

**Affiliations:** 1 Department of Biology, Dartmouth College, Hanover, New Hampshire 03755, USA; 2 Center for Environmental Health Sciences at Dartmouth, Dartmouth Medical School, Hanover NH 03755, USA; 3 Department of Pharmacology & Toxicology, Dartmouth Medical School, Hanover NH 03755, USA; 4 The School of Public and Environmental Affairs, Indiana University, Bloomington, Indiana 47405, USA; 5 The Center for Genomics and Bioinformatics, Indiana University, Bloomington, Indiana 47405, USA

## Abstract

**Background:**

Genomic research tools such as microarrays are proving to be important resources to study the complex regulation of genes that respond to environmental perturbations. A first generation cDNA microarray was developed for the environmental indicator species *Daphnia pulex*, to identify genes whose regulation is modulated following exposure to the metal stressor cadmium. Our experiments revealed interesting changes in gene transcription that suggest their biological roles and their potentially toxicological features in responding to this important environmental contaminant.

**Results:**

Our microarray identified genes reported in the literature to be regulated in response to cadmium exposure, suggested functional attributes for genes that share no sequence similarity to proteins in the public databases, and pointed to genes that are likely members of expanded gene families in the *Daphnia *genome. Genes identified on the microarray also were associated with cadmium induced phenotypes and population-level outcomes that we experimentally determined. A subset of genes regulated in response to cadmium exposure was independently validated using quantitative-realtime (Q-RT)-PCR. These microarray studies led to the discovery of three genes coding for the metal detoxication protein metallothionein (MT). The gene structures and predicted translated sequences of *D. pulex *MTs clearly place them in this gene family. Yet, they share little homology with previously characterized MTs.

**Conclusion:**

The genomic information obtained from this study represents an important first step in characterizing microarray patterns that may be diagnostic to specific environmental contaminants and give insights into their toxicological mechanisms, while also providing a practical tool for evolutionary, ecological, and toxicological functional gene discovery studies. Advances in *Daphnia *genomics will enable the further development of this species as a model organism for the environmental sciences.

## Background

Recent advances in genomics and bioinformatics are revolutionizing the process of discovering genes whose regulation has important consequences to the fitness of individuals [1,2]. These resources for genetic investigations include functional genomic tools that compare the expression profiles of thousands of genes under different conditions. Microarrays are proving to be a particularly important modern resource for identifying genes in context of their complex regulation [3]. Until recently, the application of microarrays has largely focused on studies from a select set of organisms (*Saccharomyces cerevisiae, Caenorhabditis elegans, Drosophila melanogaster, Danio rerio, Mus musculus*) that constitute some of the most well characterized laboratory models in the life sciences. While these model organisms are well suited for developmental, cellular and molecular studies – contributing a staggering amount of biological knowledge – it is difficult to relate environmental controls of gene regulation of these organisms (phenotype) to higher-level (population) responses because so little is known about their ecology. This chasm presents a challenge for toxicological genomic applications, especially those related to environmental toxicology where the goal is often to identify population and ecosystem-level responses in the context of environment change. Thus, the aim of discovering genes that are expressed as a function of ambient conditions (therefore, anchoring potential genetic biomarkers to biological/ecological functions) requires applying functional genomic approaches to keystone species with accessible natural populations and tractable ecologies, such as the ubiquitous aquatic micro-crustacean *Daphnia pulex*.

Commonly known as water fleas, *Daphnia *are familiar and ubiquitous inhabitants of ponds and lakes throughout the globe and have been the focus of study by limnologists for well over a century [4-6]. As a dominant member of the planktonic community, *Daphnia *play a central role in aquatic food webs, serving as the primary grazers of algae, bacteria and protozoans, and as the primary forage for fish. As a result, *Daphnia *are long recognized as a sentinel/indicator species in freshwater ecosystems and they are routinely used to determine the toxicity of aqueous solutions and to gauge the quality of inland waters [7]. Therefore, the gross-level responses of *Daphnia *to a number of environmental pollutants are well characterized [8], and these responses are referenced by environmental protection agencies (e.g., United States Environmental Protection Agency, Environment Canada, Organization for Economic Co-operation and Development) to define regulatory limits [9], monitor the quality of industrial and municipal effluents [10,11] and estimate the risk of environmental toxins on natural environments. For these reasons, the present study extends the "road-map" for applications of DNA microarrays to studies of non-model organisms described by Gracey et al. [12] and later by others (reviewed in [13,14]), by characterizing the response of this critical aquatic indicator species to environmental stress. These investigations utilize *D. pulex*; a freshwater crustacean species that is ubiquitous throughout North America [15], sensitive to metals and metal mixtures [16], and supported by a major genome sequencing project [17]. They also complement recent efforts by others to develop a microarray platform for *D. magna *[18]; [19]; [20].

We focus our present investigations on cadmium as a model environmental stressor, but the technologies described are pertinent to a much wider range of ecological and toxicological applications. Cadmium is a ubiquitous environmental contaminant [21]. It ranks eighth on the Agency for Toxic Substances and Disease Registry [22] list of the top 50 priority pollutants, is one of the most common contaminants found in the U.S. EPA Superfund sites [23], and is highly toxic to aquatic life. Cadmium is extremely persistent in the environment and, as a result, bioaccumulates within food webs [24]. In aquatic animals, cadmium is a potent calcium antagonist that disrupts calcium uptake and homeostasis [25-27]. Cadmium also induces oxidative stress, resulting in lipid peroxidation, damage to membranes, impaired cellular functions, and tissue damage [28]. It is a Class B metal that has ten outer shell electrons in the *d *orbital and is highly reactive, tending to preferentially form covalent bonds with S>N>P>O [29,30]. Thus, complexation with anions controls exposure in the water column [31] and ultimately toxicity in the animal [32]. Within many organisms, the major ligands for cadmium are small metal binding proteins known as metallothionein (MT) [32,33].

The metallothioneins are a family of unusual and highly conserved small cytosolic proteins, characterized by their low molecular weight (i.e., 6000–7000 Daltons), lack of secondary structure in the absence of metal ligands, absence of histidine or aromatic residues, and high cysteine content (typically 30–33% of the protein). The MTs principally bind metals from groups 1B and 2B of the Periodic Table of Elements (e.g., cadmium, copper, mercury, silver, zinc; [34,35]), and in so doing form two metal-binding domains. The metal binding properties of MT are conferred by the large number, spacing and metal coordination of the cysteine residues within the protein. It is generally recognized that the physiological functions of MT are to regulate the intra-cellular concentrations of essential metals such as copper and zinc; to activate and deactivate zinc-regulated proteins; and to scavenge free radicals [35,36]. However, MT can also confer protection from metal poisoning by binding certain free metal ions or undergoing exchange reactions with metals bound to other ligands [35,37]. The synthesis of MT is also regulated by metals with MT mRNA expression increasing in response to elevated metals in tissues [38,39]. Because of its high specificity and sensitivity for metal induction, MT levels have been successfully used to diagnose copper, zinc, and cadmium exposures in numerous studies ranging from temperate freshwater environments to tropical marine systems [40]. While MTs have been characterized in several Malacostraca crustacean species [41], with the exception of partial cDNA sequences of two MTs from *D. magna *[19] there is little sequence information available for these genes and their regulatory regions in the Branchiopoda, such as *Daphnia*.

We report a series of studies designed to test whether the exposure to sub-lethal chemical stressors results in identifiable changes in gene expression, exposing genes that respond to these conditions and providing a means of identifying potential biomarkers of response to specific exposures. Here, we developed a cDNA microarray platform for *D. pulex *to investigate differences in gene-response profiles for this aquatic indicator species following environmental perturbation by cadmium. Expression profiling successfully aided in the discovery of genes regulated in response to cadmium exposure, including the important metal biomarker, MT. Gene-response profiling provided mechanistic information that related to the observed cadmium-induced phenotypes and population-level responses. Finally, we identified and characterized the primary structure of the *D. pulex *MT sequences in context of their genomic architecture, translated sequence and phylogenetic relationship to other crustacean MTs.

## Results

### *Daphnia *response to cadmium

**Figure 1:**
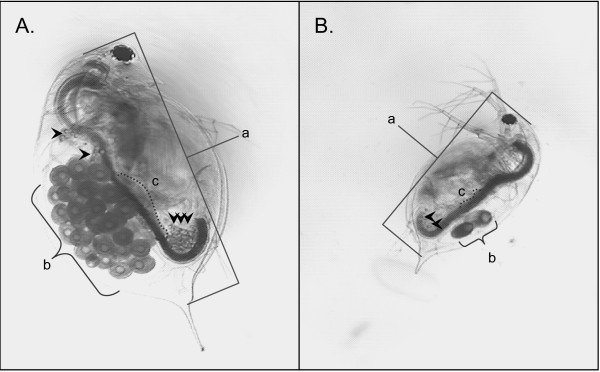
**Cadmium induced phenotype**. Representative micrographs of 21-d *Daphnia pulex *maintained under A) control conditions or B) exposed to cadmium (2.5 μg Cd/L). Images were collected at the same scale and are presented as raw image files. Differences were observed in a) body length and b) number of eggs in the brood chamber. The control animal also has a larger c) ovary and more pronounced lipid stores (represented by arrows).

To define sub-lethal exposure concentrations and better characterize cadmium-exposed phenotypes, preliminary experiments were conducted to determine the sensitivity of *D. pulex *to cadmium. For these studies, acute (48-h) toxicity tests were used to define median lethal (LC50) and sub-lethal (LC01) effects concentrations. Mortality in the control groups was less than 10% for all tests. The LC50 and LC01 values given as mean ± 95% confidence intervals were 74.6 (64.2 – 84.4) and 20.3 (9.7 – 30.1) μg/L, respectively. Demographic (i.e., life-table) experiments that included longer-term exposures (21-d) to lower cadmium concentrations (1 to 2.5 μg Cd/L) were conducted to better define sub-lethal cadmium responses. Observed effects included a decline in individual fitness parameters of size and lipid content to ovary size index, and population-level endpoints of number of clutches, cumulative reproductive rate, and per capita birth rate (Table 1). These effects are highlighted by the two representative micrographs of control and cadmium-treated animals (Figure 1), which were taken at the end of the 21-d exposure period.

**Table 1:** Effects of 21-day cadmium exposure on *Daphnia pulex*

Cadmium (μg/l)	Length (mm)	Lipid-Ovary Index^a^	Number of Clutches^a^	Total neonates^a^	Per Capita Birth Rate (b)^a^
0	2.90 ± 0.11a	3.83 ± 1.50a	5.50 ± 0.55a	118.86 ± 29.94a	5.37 ± 0.89a
0.25	2.54 ± 0.10b	1.57 ± 1.13b	4.63 ± 0.52a	65.13 ± 16.60b	2.88 ± 0.66b
0.5	2.41 ± 0.12b	1.14 ± 0.69b	4.29 ± 0.76a	51.00 ± 25.66b	2.22 ± 1.12b
1	2.26 ± 0.16b, c	1.25 ± 0.96b	2.00 ± 1.00b	8.60 ± 3.85c	0.39 ± 0.18c
1.75	2.16 ± 0.12c	1.00 ± 0.93b	2.22 ± 1.09b	13.00 ± 8.04c	0.58 ± 0.34c
2.5	2.04 ± 0.14c	1.0 ± 0.83b	1.50 ± 1.05b	5.67 ± 5.32c	0.25 ± 0.23c

^a^Values with different letters are significantly different (P < 0.05).

### Microarray results

**Figure 2:**
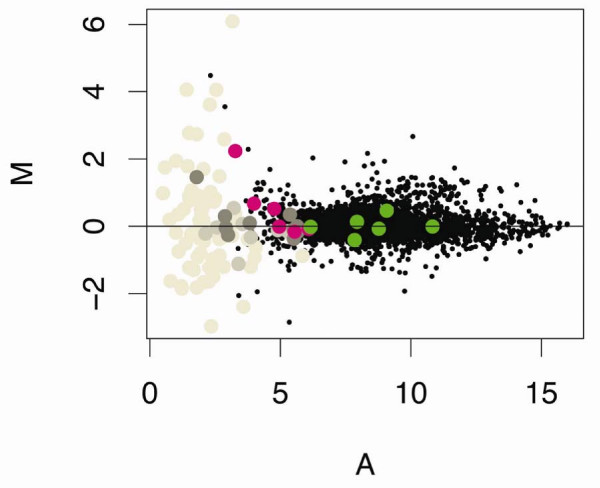
**Cadmium effects on gene expression: Buffers, blanks, and controls**. Gene expression data from control and cadmium (20 μg Cd/L for 48-h) treated *Daphnia pulex *[GEO:GSE9746]. Data were LOESS normalized; duplicate probes were averaged within LIMMA using gene-wise linear models fit to expression data, and gene expression log ratios (M = log_2 _treated/control) were plotted against log mean intensity values (A = 1/2log_2 _(treated * control). Print control elements (buffers, blanks, betaine; grey shades); negative controls (non-daphniid cDNA; pink); and positive controls (*D. pulex *cytochrome b and c, actin, and ferritin; green) are highlighted.

Microarrays were used to compare the gene-expression profiles of *D. pulex *maintained under control conditions with those exposed to 20 μg Cd/L for 48-h (i.e., sub-lethal concentration, ~LC01; [GEO:GSE9746]). These utilized RNA isolated from three independent and concurrently replicated exposures of *Daphnia *to cadmium and control conditions, applied to three replicate microarrays using a standard control vs. treated design that included dye swaps (for details see Methods). Gene expression log ratios (M = log_2 _treated/control) across the three microarrays were determined as described in Methods and plotted against log mean intensity values (A = 1/2log_2 _(treated * control) as shown in Figure 2[42]. The M-values were distributed around zero, with print control elements that contained no cDNA possessing the lowest intensity (or A values). As expected, negative control elements, which contained non-*Daphnia *cDNA also, had low A-values. However, within these two groups (i.e., print control elements, negative control elements), gene expression (or M-values) was quite varied because of the considerable noise observed with these low intensity values. Positive control elements, which contained known *D. pulex *genes (cytochrome C, cytochrome B, actin, and ferritin) distributed with other *D. pulex *elements on the MA plot.

**Figure 3:**
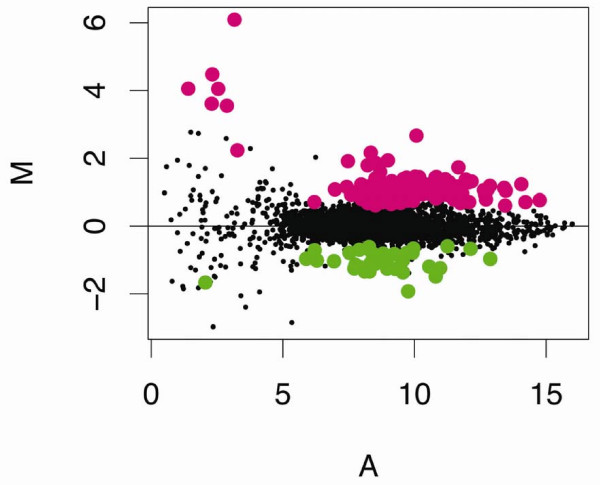
**Cadmium effects on gene expression: Regulated genes**. Gene expression data from control and cadmium (20 μg Cd/L for 48-h) treated *Daphnia pulex *[GEO:GSE9746]. Micorarray elements determined to significantly different (p ≤ 0.05) using the empirical Bayes (ebayes) method to shrink gene-wise error estimate in cadmium treated vs. control animals are highlighted (up-regulated elements in pink; down-regulated elements in green).

Empirical Bayes statistics using a p-value cut-off of 0.05 revealed 99 elements (2.9% of the array) for which expression was increased following cadmium treatment and 30 elements (1.1% of the array) for which expression decreased (Table 2, Figure 3). Of these elements, 95 were sequenced from the 5'-end. These ESTs clustered into 42 assembled sequences. Sequence analysis and alignment comparisons of these assembled sequences using the Blastx program and an expectation-value cut-off of 1 × 10^-3 ^and 33 matched amino acids [43] against the non-redundant protein sequence inventory at the NCBI identified 27 unique cDNAs with sequence similarities to known genes. These included 22 up and five down-regulated genes. Fifteen assembled sequences were unidentifiable, sharing no similarities to known genes. The difference between the number of sequenced elements identified as cadmium responsive (i.e., 95) and the total number of likely unique cDNAs (i.e., 42) was due to redundancy on the microarray. Although many of the elements whose ESTs were clustered into assembled sequences shared similar expression levels, in some cases their expression levels deviated by as much as two-fold (Table 2). The observed differences among clustered elements were even more pronounced when all sequenced elements belonging to the set of differentially regulated genes (EST cluster) were included in the analysis. The variation was not a function of differences in their intensity values, because deviations from the mean value of A were negligible (data not shown). Without further data, we were unable to verify whether some assembled sequences were composed of alternatively spliced transcripts and/or recently duplicated loci that differed in their responses to cadmium.

**Table 2:** Cadmium regulated microarray elements

*Up-regulated elements*							
Sig^a^	Seq^b^	EST Cluster	M average^c^	A average^c^	Hit Description	P Value^d^	Putative GO annotations
8	9	Contig 262	1.02 ± 0.31	10.12 ± 1.88	2-domain hemoglobin protein subunit	< 0.001	GO:0015671, GO:0005833, GO:0005344
2	16	Contig 272	0.43 ± 0.21	9.06 ± 1.50	CG30045-PA	< 0.001	GO:0042302
1	1	Singlet 433	0.71	12.08	CG6305-PA	0.062	GO:0042302
1	1	Singlet 498	1.02	9.15	chitinase-1	0.028	GO:0008061, GO:0006030, GO:0004568, GO:0005576
3	4	Contig 213	0.82 ± 0.12	9.07 ± 0.53	chitinase-2	< 0.001	GO:0008061, GO:0006030, GO:0004568, GO:0005576
1	3	Contig 218	0.93 ± 0.19	9.64 ± 1.77	chitotriosidase	0.005	GO:0008061, GO:0006030, GO:0016798, GO:0005576
1	3	Contig 209	0.41 ± 0.17	12.21 ± 1.48	CUO6 BLACRCuticle protein 6 (BcNCP14.9)	0.06	GO:0042302
4	6	Contig 241	1.07 ± 0.31	10.76 ± 2.07	cuticle protein-1	0.018	GO:0042302
5	8	Contig 257	1.03 ± 0.33	9.96 ± 1.54	cuticle protein-2	< 0.001	GO:0042302
13	28	Contig 273	0.91 ± 0.32	10.05 ± 1.65	cuticle protein-3	< 0.001	GO:0042302
1	3	Contig 149	0.72 ± 0.28	8.55 ± 0.92	cuticle protein-4	0.011	GO:0042302
5	8	Contig 261	0.88 ± 0.30	9.87 ± 1.42	cuticle protein-5	< 0.001	GO:0042302
1	1	Singlet 15	1.23	14.06	ERGA6350	0.009	
1	1	Singlet 469	0.80	7.99	helix-loop-helix transcription factor	0.055	GO:0006355, GO:0003677
1	1	Contig 21	1.45	10.03	hemoglobin-1	0.003	GO:0015671, GO:0005833, GO:0005344
1	1	Contig 16	1.08	9.81	hemoglobin-2	< 0.001	GO:0015671, GO:0005833, GO:0005344
1	1	Singlet 65	2.16	8.34	Hypothetical protein CBG14247	< 0.001	
4	4	Contig 221	1.98 ± 0.47	8.88 ± 0.82	Metallothionein	< 0.001	GO:0046872, GO:0006875
1	5	Contig 220	0.31 ± 0.49	8.95 ± 0.62	myosin 2 light chain	0.08	GO:0005509
2	4	Contig 227	0.70 ± 0.04	12.59 ± 2.37	OPSC1 HEMSACompound eye opsin BCRH1	0.036	GO:0007602, GO:0016021, GO:0001584, GO:0007186
1	1	Singlet 251	1.18	10.90	PREDICTED: similar to chitinase	0.019	GO:0008061, GO:0006030, GO:0016798, GO:0005576
1	1	Singlet 459	1.15	7.42	PREDICTED: similar to glutathione S-transferase	0.021	GO:0016740
1	1	Singlet 1	1.94	8.99	Unknown EST-1	0.009	
3	3	Contig 232	1.14 ± 037	8.27 ± 0.99	Unknown EST-2	< 0.001	
7	8	Contig 253	1.05 ± 0.44	9.99 ± 1.38	Unknown EST-3	< 0.001	
1	1	Singlet 166	0.87	8.20	Unknown EST-4	0.048	
1	1	Singlet 64	0.75	9.95	Unknown EST-5	0.059	
1	1	Singlet 49	0.74	8.77	Unknown EST-6	0.067	
1	5	Contig 237	0.33 ± 0.21	10.11 ± 1.55	Unknown EST-7	0.073	
1	1	Singlet 172	0.68	8.23	Unknown EST-8	0.071	
24					Unsequenced		

*Down-regulated elements*							
Sig^a^	Seq^b^	EST Cluster	M average^c^	A average^c^	Hit Description	P Value^d^	Putative GO annotations

1	2	Contig 135	-0.36 ± 0.66	9.28 ± 0.08	carboxypeptidase A1	0.068	GO:0006508, GO:0004182
1	1	Singlet 97	-1.04	6.94	endo-1,4-mannanase	0.009	GO:0016985, GO:0000272
1	3	Contig 202	-0.38 ± 0.36	8.39 ± 1.02	PREDICTED: similar to CG31997-PA	0.036	
2	2	Contig 83	-1.29 ± 0.06	8.00 ± 0.40	PREDICTED: similar to monooxygenase	0.001	
1	3	Contig 162	-0.37 ± 0.30	8.98 ± 1.04	ribosomal protein S11-2	0.02	GO:0006412, GO:0003735, GO:0005840
1	2	Contig 191	-0.61 ± 0.27	10.76 ± 3.35	Unknown EST-1	0.019	
2	4	Contig 212	-0.72 ± 0.38	8.77 ± 0.97	Unknown EST-2	< 0.001	
4	5	Contig 229	-1.32 ± 0.11	9.96 ± 0.90	Unknown EST-3	< 0.001	
4	7	Contig 249	-1.08 ± 0.27	8.13 ± 0.98	Unknown EST-4	< 0.001	
1	2	Contig 56	-0.41 ± 0.36	9.25 ± 0.99	Unknown EST-5	0.056	
1	1	Contig 71	-0.71	6.21	Unknown EST-6	0.027	
1	1	Singlet 79	-1.08	8.90	Unknown EST-7	0.014	
10					Unsequenced		

^a^Number of microarray elements determined to be significant on the array using the empirical Bayes method to shrink the gene-wise error estimate in cadmium treated vs. control *Daphnia pulex *(P < 0.05). control *Daphnia pulex *(P ≤ 0.05).

^b^Number of microarray elements selected for sequencing based on cadmium response determined in the current study; Eads et al., in review; and Colbourne et al., in review.

^c^Mean value (± SD) based on all sequenced microarray elements.

^d^Permutation tests were performed for clones sharing common putative annotations. These tests included 1000 simulations using the mean expression values to determine the Probability of obtaining the M average for an EST cluster.

### Functional attributes of responsive genes

**Figure 4:**
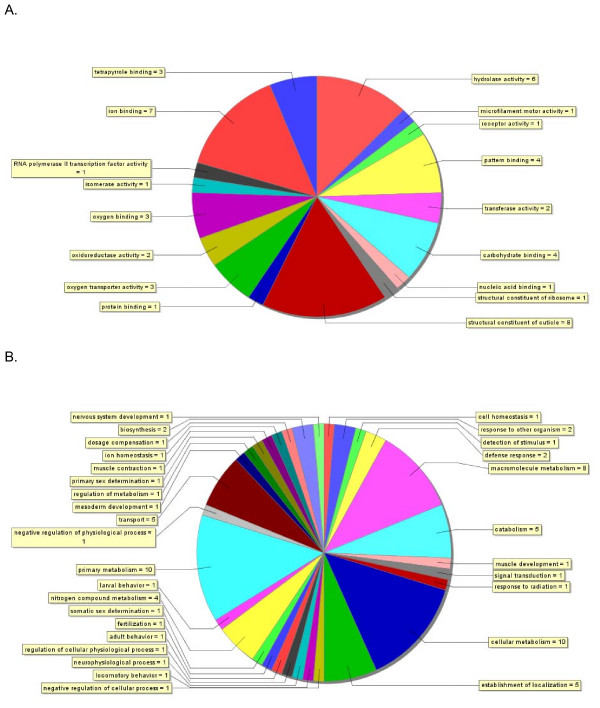
**Annotations of genes responding to cadmium**. The distribution of gene annotations for the list of 45 *Daphnia pulex *genes (EST clusters) responding to cadmium treatment on the microarray based on results from Blastx searches against the NCBI non-redundant protein database. (A) The assignment of 49 annotations of molecular function to 26 genes from level 3 of the Gene Ontology. (B) The assignment of 74 annotations of biological process to 17 genes from level 4 of the Gene Ontology. Blastx queries recorded the best 5 matches with an E-value threshold of 1 × 10^-3 ^and a minimal value of 33 aligned amino acids. Gene Ontology (GO) terms were assigned to genes using Blast2GO [41] with the following configurations: Pre-eValue-hit filter 1 × 10^-3^; Pre-similarity-hit filter 2; Annotation cut-off 35; GO weight 5.

The molecular functions and biological processes of *Daphnia *genes responding to the cadmium treatment were investigated based on the putative assignment of Gene Ontology (GO) terms using Blast2GO [44]. Their functions spanned a defined set of gene ontologies (Figure 4a). A total of 23 genes (i.e. EST clusters) were assigned 49 molecular functional terms from the third level of the GO. The predominant terms included structural constituent of the cuticle (16%) and ion binding (14%). Indeed, 14 genes were altogether annotated as binding proteins, of which seven were annotated as metal ion binding (iron, calcium), four as carbohydrate (also listed as protein or chitin) binding proteins, three specifically as oxygen binding proteins (hemoglobins, also listed as tetrapyrrole binding proteins), one protein binding and one nucleic acid binding protein. This last gene (Singlet 469) is a transcriptional regulator whose best match to a *Drosophila *protein is *Similar to Deadpan *(FlyBase ID: FBgn0032741). Six genes also function as hydrolases (Figure 4a).

As well, a total of 17 genes were assigned 74 biological process terms from the fourth level of the GO. The majority of the terms were for metabolic processes ascribed to 11 genes, which included cellular metabolism (14%), primary metabolism (14%), macromolecule metabolism (11%), catabolism (7%), nitrogen compound metabolism (5%), biosynthesis (3%) and regulation of metabolism (Figure 4b). Remarkably, a total of four genes were attributed roles in chitin metabolism, including chitinases and chitotriosidase. Another predominant biological process was related to the localization of cellular components (establishment of localization, transport, protein localization), which were ascribed to five genes. Three of these sequences coded for genes involved in oxygen transport (hemoglobins). A ferritin gene was also differentially regulated during the experiment, which binds iron and is involved in iron regulation and storage [45,46] and was ascribed the functions of cell and ion homeostasis. Finally, the remaining differentially regulated genes with annotations were likely involved in cell communication (such as signal transduction), development, and physiological processes (such as defence response) that specify a reaction to external stimuli, or stress.

### Confirmation of microarray result

**Figure 5:**
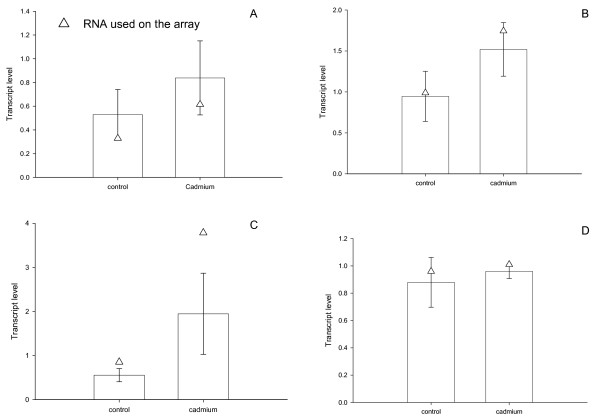
**Confirmation of array results**. Quantitative-real time (Q-RT)-PCR was used to confirm a subset of genes identified with the microarrays to be regulated in response to cadmium; A) cuticle protein-2, Contig 257, B) 2-domain haemoglobin protein subunit, Contig 262, C) metallothionein, Contig 221) and one gene for which expression was not altered D) serine-threonine kinase, Contig 274 (Table 2). Sequences for primer pairs and probes are provided in Table 3. Expression levels were validated using aliquots sub-sampled from pools of RNA that were used for microarray analysis (technical validation, open triangles) and RNA collected from repeated, independent exposures (biological validation, open bars, mean ± SD, n = 5).

Following sequence analysis, expression levels of a subset of genes identified as cadmium-responsive (Table 2) were measured to validate microarray output. This included three cadmium responsive genes (i.e., cuticle protein-2, Contig 257; 2-domain haemoglobin protein subunit, Contig 262; metallothionein, Contig 221) and one gene for which expression was not altered (i.e., serine-threonine kinase, Contig 274). Expression levels were confirmed by Q-RT-PCR (Table 3) using aliquots sub-sampled from pools of RNA that were used for microarray analysis (technical validation) and RNA collected from repeated, independent biological exposures (biological validation). In all instances, expression levels measured by Q-RT-PCR agreed with microarray output (Figure 5) both in terms of direction (M) and relative magnitude (A) of the response.

**Table 3:** Real-time PCR primer pairs

*Contig*	*Gene*	*sense*	*antisense*	*TaqMan probe*	*RT primer*
*257*	Cutilcle protein	CGTCGCCGATGTGAAATAC	AAGAAGAACCTTTGTGATAGGAATC	GGATATGCCAAGTACCCCGAGT	GGCATCGTATTTTGGA
*262*	Hemoglobin	TTCAAAGCCAAACCCGAAGC	TTGGCAAATCCGTAATGGACA	AGAAGCTCTTTTCGGAATTCGCCAACG	AGCGTTCAGGAAATCGT
*221*	Metallothionein	AAACTACCCAACGGAATCAACAT	CAGTTGGGTCCGCATTTG	CCACACGAGCATTTACCTTGGCAAC	
*274*	STK^a^	TTTTTAACAGAACCATCTTTGTCCAA	GACATAGTTTTTCAACATTCCTTCACAG	GTGTAAGTACGAGTTAAAGAAATTATCAGCCATC	CTGATACACAAGGTACGATAA

^a^Serine threonine kinase

### Characterization of *Daphnia *metallothioneins

**Figure 6:**
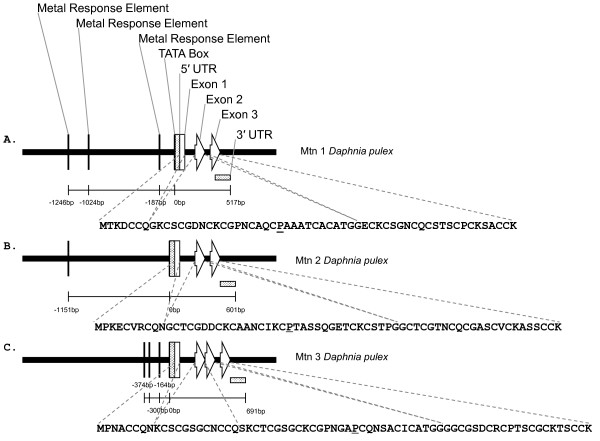
**Metallothionein gene models**. *Daphnia pulex *metallothionein gene models. A) Dpu Mtn1 [GenBank:EU307302], B) Dpu Mtn2 [GenBank:EU307303], and C) Dpu Mtn3 [GenBank:EU307304].

Given its utility as a biomarker, we further characterized the putative *D. pulex *MT, which represents one of the first MT sequences identified from a non-malacostracan crustacean species. A putative MT was identified by the microarray following the sequencing of probes whose expression was up-regulated by cadmium exposure (Figure 6a). A sequence similarity search against the NCBI protein database using Blastx revealed little similarity to other MTs. The closest match at the time was directed to MT from the giant keyhole limpet, *Megathura crenulata *(E-value = 0.036), but this is well below 1 × 10^-5 ^E-value considered to be significant [43]. However, the translated sequence revealed a 59 amino acid protein of high cysteine content (30.5%) that contained no aromatic amino acids or histidine residues, unique features that are hallmarks of MT. The 18 cysteine residues were arrayed in characteristic Cys-xaa-yaa-Cys (1), Cys-x-Cys (6), and Cys-Cys (2) motifs, establishing it as a class 1 MT (Fig. 5; [47]). Nevertheless, the translated amino acid sequence was still quite diverged from other crustacean MTs (~30% similarity), including differences in the common pattern observed at the N terminus, P- [GD]-P-C-C-x(3,4)-C-x-C [48]. Despite this lack of similarity, the number of cysteine residues was identical to other crustacean MTs and these showed a high degree of conservation when aligned with sequences reported for other arthropods (Table 4). Based on amino-acid alignments with characterized MTs, the *D. pulex *MT formed two coordinative domains (α, C-terminus; β, N-terminus), hinging with the proline residue at position 20 (Fig. 6a, Dpu Mtn1). It should be noted that during the preparation of this manuscript, an MT gene transcript and translated amino acid sequence was reported for another daphniid, *D. magna *[19].

**Table 4:** Amino acid alignment of three *D. pulex *metallothionein genes against those from other crustaceans (decapods) and from selected insects^a^.

Protein domains	|-------------- Beta --------------| |------------------- Alpha --------------------|
Mtn1 *Anopheles gambiae*	MPCKCCGN-D**C**K**C**TSG---**C**GSGQP**C**AT---D**C**K**C**ACASGGCKEKS------------------------------GG**CC**GK--
Mtn2 *Anopheles gambiae*	MPCKT**C**VA-D**C**K**C**TSP--N**C**GAG**C**G**C**ES---R**C**T**C**PCKDGAK----------------------------------EG**CC**K---
MtnA *Drosophila melanogaster*	MPCP-**C**GS-G**C**K**C**ASQ--ATKGS**C**N**C**GS---D**C**K**C**G---------------------------------GDKKSA-CG**C**SE---
MtnB *Drosophila melanogaster*	MVCKG**C**GT-N**C**Q**C**SAQ--K**C**GDN**C**A**C**NK---D**C**Q**C**VCKNGPK----------------------------------DQ**CC**SNK-
MtnC *Drosophila melanogaster*	MVCKG**C**GT-N**C**K**C**QDT--K**C**GDN**C**A**C**NQ---D**C**K**C**VCKNGPK----------------------------------DQ**CC**KSK-
MtnD *Drosophila melanogaster*	MGCKA**C**GT-N**C**Q**C**SAT--K**C**GDN**C**A**C**SQ---Q**C**Q**C**SCKNGPK----------------------------------DK**CC**STKN
CuMtn2 *Callinectes sapidus *CRAB	MPCG-**C**GT-S**C**K**C**GSGKCC**C**GST**C**N**C**TTCPSKQS**C**SCNDGACGSAC--------------QCKTSCCCGADCK---CSP**C**PMK-
Mtn1 *Callinectes sapidus *CRAB	MPGPC**C**ND-K**C**V**C**QEG--G**C**KAG**C**Q**C**TS----**C**R**C**S-PCQKCTSGC--------------KCATKEECSKTCTKP-CS**CC**PK--
Mtn2 *Callinectes sapidus *CRAB	MPDPC**C**ND-K**C**E**C**KEG--E**C**KTG**C**K**C**KS----**C**R**C**P-PCDKCSSEC--------------KCTSKEECSKTCSKP-CS**CC**P---
Mtn1 *Scylla serrata *CRAB	-PGPC**C**ND-K**C**V**C**KEG--G**C**KEG**C**Q**C**TS----**C**R**C**S-PCEKCSSGC--------------KCANKEECSKTCSKA-CS**CC**PT--
Mtn2 *Scylla serrata *CRAB	MPDPC**C**ID-K**C**D**C**KEG--E**C**KTG**C**K**C**TS----**C**R**C**P-PCEQCSSGC--------------KCANKEDCRKTCSKP-CS**CC**P---
Mtn1 *Potamon potamios *CRAB	-PDPC**C**AEGT**C**E**C**EEG--K**C**KAG**C**K**C**TS----**C**R**C**S-PCEKCTSEC--------------ECKSKEECAKNCTKP-CS**CC**P---
Mtn *Carcinus maenas *CRAB	MPDPC**C**ID-K**C**E**C**KEG--G**C**KAG**C**K**C**TS----**C**R**C**T-PCEKCSSGC--------------KCTTKEDCCKTCTKP-CS**CC**P---
Mtn *Eriocheir sinensis *CRAB	MPDPC**C**ND-K**C**E**C**KEG--K**C**EAG**C**K**C**TS----**C**R**C**P-PCEKCSSGC--------------KCGSKEDCCKTCSKP-CS**CC**P---
Mtn *Portunus pelagicus *CRAB	MPDPC**C**ID-K**C**E**C**KEG--K**C**EAG**C**K**C**TS----**C**R**C**P-PCEKCSSGC--------------KCGSKEDCCKTCSKP-CS**CC**P---
Mtn *Panulirus argus *LOBSTER	MPGPC**C**ID-K**C**E**C**AEG--K**C**KSG**C**Q**C**KS----**C**T**C**STPCDKCTTAC--------------CCSTKEECASKCTKP-CK**CC**P---
Mtn *Homarus americanus *LOBSTER	MPGPC**C**KD-K**C**E**C**AEG--G**C**KTG**C**K**C**TS----**C**R**C**A-PCEKCTSGC--------------KCPSKDECAKTCSKP-CK**CC**P---
Mtn *Astacus astacus *CRAYFISH	MPGPC**C**ND-V**C**E**C**AAG--G**C**KTG**C**V**C**TS----**C**R**C**S-PCDKCTSGC--------------KCPSKEECAKTCSKP-CE**CC**P---
Mtn1 *Daphnia pulex *cDNA	MTKD--------------C**C**QGK**C**S**C**GD---N**C**K**C**GPNCAQCPAAA----TCACATGGECKCSGNCQCSTSCPCK-SA**CC**K---
Mtn2 *Daphnia pulex *cDNA	MPKECV------------R**C**QNG**C**T**C**GD---D**C**K**C**AANCIKCPTASSQGETCKCSTPGGCTCGTNCQCGASCVCKASS**CC**K---
Mtn3 *Daphnia pulex *cDNA	MPNAC**C**QN-K**C**S**C**GSGCNC**C**QSK**C**T**C**GS---G**C**K**C**GPNGAPCQNSA-----CICATGGG-GCGSDCRCPTSCGCK-TS**CC**K---
Mtn3 *Daphnia magna *cDNA¥	MPNVC**C**QN-N**C**S**C**GNG**C**TC**C**QSK**C**T**C**GS---G**C**K**C**GPNGAPCQNSA-----CICATGGG-GCGSDCRCPVSCGCK-TS**CC**K---
Cysteine residues	0 00 0 0 0 0 0 0 1 1 1 1 1 1 1 1 1 1 2 2 2 22
	1 23 4 5 6 7 8 9 0 1 2 3 4 5 6 7 8 9 0 1 2 34
Metal binding	** * * * * * * * * * * * * * * **

^a^Cysteine residues are **bold **for sites that are conserved in crustacean and insect genes (residues 3–10, 23, 24) and underlined when residues are clade-specific. Assuming a correct alignment, residues 2, 12, 13, 17, 19 and 20 are conserved in all (or most) of the crustacean genes, whereas residues 14 and 22 are conserved in the decapod genes. The residues 15, 16, 18 and 21 are exclusively found in the Daphnia genes (representing branchiopods) and based on the limited sampling of insect genes (from two dipterans), residues 1 and 11 are excluded in the crustacean genes. Domain partitions and metal bind residues (*) are based on experimental evidence from decapod proteins (Valls et al. [41]). ¥ Poynton et al. [19] provides full translation.

Following this preliminary identification of the MT gene, further cDNA sequences were recognized from within a growing *D. pulex *cDNA sequence database [49], which was created as part of the annotation process for the *Daphnia *genome sequencing project (Colbourne et al., in prep). Using tBlastx, a total of 9 assembled EST clusters were discovered, representing transcripts from three genomic loci. Using Blastn, the regions within genomic sequence scaffolds were identified that matched the metallothionein cDNA sequences (Figure 6). The locus corresponding to the cDNA identified on the array, called Dpu Mtn1 [GenBank:EU307302] (Fig 6a), consists of three exons (86 bp, 90 bp, 168 bp) and two introns (107 bp, 76 bp). The 5' untranslated region (UTR, at least 64 bp) is fully contained within the first exon, while the 3' untranslated region consisted of 103 bases. The region upstream of the 5'UTR contains three putative regulatory elements showing exact sequence similarity with the core consensus sequence of metal responsive elements (TGCRCNCS, where R is A or G and S is C or G; [50,51]). This flanking region also contains a TATA box 20 bases upstream of the putative transcriptional start site. Within the 5'UTR, the area immediately upstream of the ATG codon agrees with Kozack's rules of ribosome binding. The eukaryotic polyadenylation signal (AATAAA) is found in the 3'UTR. The second identified locus called Dpu Mtn2 [GenBank:EU307303] (Fig 6b) encodes a protein that consists of 66 amino acids. Like the first locus, this gene is composed of three exons (70 bp, 99 bp, 223 bp) and two introns (141 bp, 70 bp). Its 5'UTR is at least 42 bases and contained within the first exon, while the 3'UTR consists of 152 bases. The region upstream of the 5'UTR contains a single putative metal responsive elements (-1151 bp). Finally, the third locus called Dpu Mtn3 [GenBank:EU307304] (Fig 6c) codes for a protein that consists of 70 amino acids that is almost identical to the amino acid sequence provided by Poynton et al. [19] for *D. magna *(Table 4). The 5'UTR of Dpu Mtn3 is at least 66 bases in length and the 3'UTR consists of 163 bases. Three putative metal response elements are found upstream of the 5'UTR (-164, -300, -374 bp). Unlike the other daphniid MTs, this gene is composed of four exons (88 bp, 39 bp, 84 bp, 232 bp) and three introns (131 bp, 59 bp 62 bp).

### Phylogenetic analysis of crustacean metallothioneins

The amino acid sequences of *D. pulex *MTs were aligned and compared to 19 amino acid sequences from 13 other crustaceans, including the recently identified *D. magna *sequence, and two insects to determine the phylogeny of the protein within Crustacea (Table 4). These comparisons were made difficult by the lack of sequence conservation among the arthropod MTs (12.7% similarity). The greatest sequence divergence was found among the insect genes obtained from fully sequenced genomes, which averaged 72.3%. By contrast, the three *D. pulex *genes averaged 56.1% sequence divergence. This divergence was measured from genes that were also identified from a well characterized genome. Considering that the average divergence among the other entire crustacean MTs was only 27.4%, most of the characterized malacostracan genes were likely homologues or recent duplicates among their representative genomes. Clearly, further genome-wide investigations among the Crustacea will uncover paralogous loci that will broaden the phylogenetic account of this protein family.

While little similarity was observed between primary amino acid sequences, there was a great deal of conservation of the cysteine residues. The MTs contained an average of 30.4% cysteines; crustacean genes contained 3.5% more cysteines than the representative insects. This conservation translates into structural homologies that coordinate the disulfides within the protein domains, which are responsible for the molecules' unique metal binding properties. From the amino acid alignment, 10 cysteine residues were found to be largely preserved in both the insect and crustacean genes (Table 4). Some exceptions were observed. In particular, our alignment suggested that two of the three *Daphnia *genes lost the conserved residues 3–5. The two known copper-binding MTs also showed a single cysteine instead of two at the C-terminus of the proteins. A second class of conserved residues was composed of cysteines that are known to bind metals in malacostracans. Yet, these cysteines are generally absent in the insects. Here, most of the *Daphnia *genes shared six of the eight residues. However, they all lacked residues 14 and 22. The *Daphnia *MTs contained a third class of conserved residues, whose four members are exclusive to this set of proteins (Table 4). Finally, the insects' α-domains are substantially reduced relative to the Crustacea. As a result, only two cysteine sites form the fourth class of residues, which are largely missing from the aligned proteins of the other taxa.

**Figure 7:**
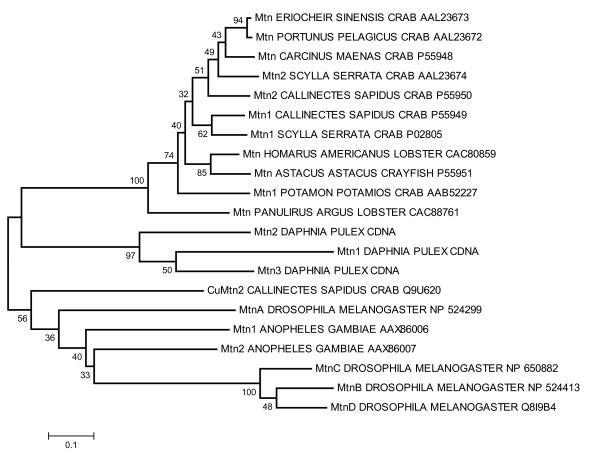
**Phylogeny of crustacean metallothioneins**. Neighbor-Joining tree constructed from an amino acid alignment of the three *Daphnia pulex *metallothionein proteins plus 18 sequences from 11 selected insect and crustacean taxa using the E-INS-I algorithm implemented by MAFFT [92]. Gaps within alignments were ignored in pairwise comparisons of the sequences and the genetic distances were corrected by the Poisson distribution model. The NCBI accession numbers are listed beside each taxonomic gene designation. Percent bootstrap support for nodes are shown, which are derived from 1000 pseudo-replication of the data. The *Daphnia *sequences were deposited at GenBank under the accession numbers Dpu Mtn1 [GenBank:EU307302]; Dpu Mtn2 [GenBank:EU307303]; and Dpu Mtn3 [GenBank:EU307304].

From this alignment, phylogenetic trees were constructed by neighbor-joining and maximum likelihood methods. The maximum likelihood tree was inferred from 77.6% fully resolved and 5.9% partly resolved quartets. Although this latter tree was not completely resolved (tree not shown), the results from both methods were congruent and showed three strongly supported monophyletic groupings with support values at nodes > 95% (Figure 7). The first group consisted of the cadmium-binding malacostracan MTs, including the presumed recent gene duplicates in the *Callinectes *and *Scylla *genomes. Both trees failed to place all the crab genes above a common node at the exclusion of the lobster and crayfish loci. In fact, a weakly supported node united the *Potamon *and *Panulirus *genes in the maximum likelihood tree (57%). The second group consisted of the three *Daphnia *MTs stemming from the base of the branch leading to the cadmium-binding malacostracan genes. Although this monophyletic grouping was strongly supported, 31% of the maximum likelihood bipartitions united Dp Mtn1 with Mtn2 and placed Dp Mtn3 at its base. The third group was composed of three *D. melanogaster *MTs, which lacked the *Drosophila *MtnA gene to form an intra-genomic clade, as seen in *Daphnia*. The excluded gene was instead placed at an earlier branch point of the insect clade, which included two intervening *Anopheles *genes (Figure 7). Finally, the copper-specific *Callinectes *MT was positioned at the root of the insect clade, along with the *Drosophila *copper thionein MtnA locus and the *Anopheles *Mtn1 gene. The relative positions of these genes were unresolved on the maximum likelihood tree, whereas 59% of the bipartitions supported the placement of the *Anopheles *Mtn2 gene at the base of the group 3 *Drosophila *genes.

## Discussion

In this paper we describe studies with newly developed *D. pulex *cDNA microarrays investigating the expressed mRNA responses of daphniids to the environmental stressor cadmium. Although microarrays have been successfully used to identify patterns of genes responding to metal exposure in organisms for which there is an abundant amount of sequence information [52,53], it is often difficult to relate the identified genes to expressed phenotypes. These challenges exist in part because the environmental contributions to phenotype in most common laboratory species are poorly understood. However, as noted by Gracey et al., [12], abundant sequence data are not a pre-requisite for gene-expression profiling. Thus, one aim of the present study was to develop and apply microarray technologies to the ecologically tractable aquatic micro-crustacean, *D. pulex*. These studies revealed patterns of gene response that provide insight into the biological and potentially toxicological responses to this important environmental contaminant. The data emphasize the potential of this approach as a tool for discovering genes regulated by an environmental stressor, but in a species for which gene expression profiles can be interpreted in context of individual and population-level effects. Associations with cadmium induced phenotypes, identification of genes reported in the literature to be regulated following cadmium exposure, and independent validations via Q-RT-PCR provided support for their utility in this role. In addition, these studies led to the discovery *D. pulex *MT mRNAs and gene sequences, providing an important new biomarker for *Daphnia *studies, while also extending the phylogeny of this class of genes. The successful identification of the metal binding protein, MT, is highlighted, not only due to its critical biological functions, but also because the blind microarray approach provided advantages over cloning methods that require significant sequence similarity to isolate genes.

We identified the acute (48-h) and chronic (21-d) responses of our *D. pulex *isolate to cadmium to better define sub-lethal exposure concentrations and cadmium-induced phenotypes. The present study is in agreement with those of others [16,21] that indicate *D. pulex *is one of the most acutely sensitive aquatic species to cadmium [54]. Reported LC50 values are comparable to data published by others (i.e., range from 46 to 90 μg Cd/L; [16]). As others have noted, chronic exposure of *D. pulex *to cadmium reduced individual fitness parameters, such as length and lipid-ovary indices, and inhibited population endpoints, such as number of clutches, cumulative reproduction, and per capita birth rate (Table 1; [55,56]). These endpoints have been quantitatively linked to population success [57].

For example, storage of lipids, vitellogenisis, and ecdysis (i.e., molt) are physiologically and chronologically associated with reproductive success and survivorship [57]. Adipocytes are incorporated into the ovary during egg development providing energy for reproduction. Parthenogenetic embryos are then transferred into the brood chamber that is contained within the carapace for further development and the process ends with newborn *Daphnia *released into the water column during the molt. Also, molt directly relates to growth, as *Daphnia *size is constrained by the carapace. Therefore, decreased length, lowered lipid-ovary indices, and fewer clutches comprised of fewer offspring can be explained by impaired vitellogenisis and/or ecdysis. Cadmium has been reported by others to inhibit ecdysis [58] and vitellogenisis [59,60] in arthropods.

Cadmium may also decrease daphniid size by limiting calcium intake. Calcium content is directly related to the mass of the carapace and, subsequently size [61] and cadmium is known to impair calcium uptake and metabolism via substitution [62]. However, several studies have shown that while the period of increased calcium flux during molt can result in increased cadmium uptake [63,64], cadmium does not affect calcium accumulation or content [64]. Yet interestingly, our studies revealed two cDNAs that responded to cadmium that represent putative calcium binding proteins (singlet 97 and contig 220), suggesting that cadmium may affect calcium regulatory pathways in more subtle ways that contribute to its overall effects.

The present study is one of the first to apply microarray technologies to *D. pulex*. These "blind arrays" identified 99 elements for which gene-expression was positively regulated and 30 elements that were negatively regulated by cadmium. However, sequence analysis revealed that these probes included several redundant features. When they were assembled into EST contigs to isolate unique features, 30 up-regulated and 12 down-regulated genes were identified (Table 2). Given the redundancy that existed on the array and the 'blind' approach-employed, the observation of redundant, cadmium-regulated elements (e.g., cuticle protein) was reassuring. In addition, the number of unique elements regulated following exposure to cadmium only represents a very small percentage (~2%) of the estimated 1,550 unique elements on the array, which is similar to other microarray studies involving sub-toxic cadmium exposures (2%, [52]; 1%, [65]; 1–3%, [66] and as suggested by Andrew *et al.*[52] is consistent with expectations for low, non-toxic exposures that tend to induce specific pathways.

The *D. pulex *microarrays and their genomics database have proven to be insightful experimental tools for identifying genes regulated by cadmium. Yet, relating the gene sequence to putative gene function is made difficult by the excessively large phylogenetic distances between *Daphnia *and its closest relatives among the classical genomic model systems. For example, the best model system to *Daphnia *with extensive functional genomic information is *Drosophila*, which last shared a common ancestor some 600 M years ago. There are also few ESTs that share sequence conservation with proteins in public databases; crustacean proteins represent only 0.1% of 6.9 million records in the NCBI taxonomic database. Of the 42 unique cadmium-regulated genes identified, only three were homologous to known *Daphnia *genes. Likewise, almost 36% of the elements identified were ESTs showing no sequence similarity with known proteins. Gracey *et al.*[12] reported similar successes with 'blind' microarrays, as approximately 40% of identified elements were unidentifiable ESTs. However, as these authors noted, novel ESTs may represent untranslated regions of previously identified genes or multiple distinct regions of unknown genes. These possibilities cannot be dismissed; we estimate that 1/4 of the orphan genes are unknown because of sequences not extending into recognizable functional domains of the gene [67]. The remaining 27 elements regulated in response to cadmium exposure on the microarray were putatively identified by similarity with known proteins.

The genes regulated in response to cadmium exposure identified in these experiments provided several measures of the utility of this approach for gene discovery and its reliability in identifying biologically sensible patterns of gene regulation. For example, many of the genes responding to cadmium exposure were part of common physiological pathways (i.e., ecdysis, metal detoxication) and few were indicative of general stress response (e.g., heat shock proteins, heme-oxidase) or overt cellular toxicity (e.g., housekeeping genes).

**Figure 8:**
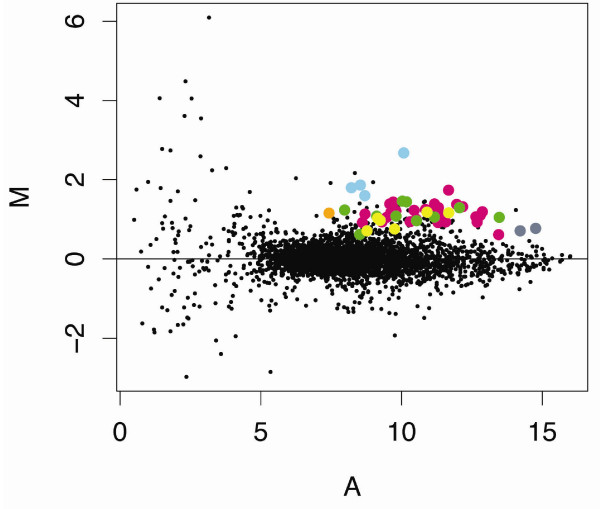
**Cadmium effects on gene expression: literature reports**. Gene expression data from control and cadmium (20 μg Cd/L for 48-h) treated *Daphnia pulex *[GEO:GSE9746]. Micorarray elements, which are known from the literature to be regulated by cadmium, are highlighted: Cuticle protein, pink; Hemoglobin, green; Metallothionein, blue; Ferritin, orange; Chitinase, yellow; Opsin, grey.

Ecdysteroid-responsive genes and other molt related genes that included chitinases 1 and 2, chitotriosidase, BcNCP 14.9, cuticle proteins 1 through 5, and singlet 251, comprised the majority of genes regulated in response to cadmium exposure (Figure 8, Table 2). This finding is critical given that altered expression of these genes, which are associated with the exoskeleton, provides a direct mechanistic link with demographic endpoints and individual fitness parameters that indicated ecdysis (i.e., molt) is impaired in *Daphnia *following cadmium exposure (Figure 1, Table 1). Deep sequencing of cDNA libraries constructed from RNA isolated from cadmium-exposed *D. pulex *provided support of these findings (Colbourne JK, personal communication). Cadmium derived libraries were enriched with genes that were identified as structural constituents of the cuticle. This observation suggests that ecdysis and molt related regulatory pathways are generally influenced by cadmium, which has been observed by others [63,64]. It is interesting that short-term exposures to cadmium of the *Daphnia *used in the array experiment provided patterns of response that were meaningful in interpreting demographic experiments that involved longer-term exposures to lower cadmium concentrations. This observation raises the possibility that an approach such as this could provide biomarkers that serve as early indicators of chronic exposures, which is an area we are currently exploring, but beyond the scope of the current manuscript.

These experiments also identified altered expression-levels of genes known to be regulated by cadmium (Figure 8). These included the metal binding protein, MT, which plays a central role in cadmium detoxication and is known to be induced by cadmium [19,41,68]. In addition, the microarrays identified a gene responsible for oxygen transport and iron metabolism in *Daphnia*, hemoglobin [69]. Expression of hemoglobin is known to be induced by anoxia, limiting iron supplies, and cadmium [19,70,71]; unpublished results). As discussed above, the arrays also identified structural components of the cuticle and associated regulatory components (i.e., chitinase) and chitinase activity has been shown to positively correlate with cadmium concentrations in previous studies [19,72]. Likewise, opsin was found to be regulated in response to cadmium on the microarrays, and cadmium has been shown to interfere with photo-behavior in daphniids, which is a complex genetically linked trait involving kairomone signalling, photoreceptor detection (e.g., opsin, [73]), and negative phototaxis [74,75]. At the cellular-level cadmium is known to induce oxidative stress [28]. Glutathione S-transferase (GST), a phase II enzyme that catalyses the conjugation of reduced glutathione with electrophilic compounds, is an important antioxidant defence enzyme that works to detoxify the products of oxidative stress [76]and was up-regulated on the array. GST gene transcription [19] and enzyme activity [77] have been shown by others to increase following exposure to cadmium.

Metallothionein induction is the principal adaptive response associated with organism survival during exposure to elevated cadmium concentrations [41,78]. The discovery of *D. pulex *MTs highlight the advantages of this approach in identifying genes regulated by cadmium. While the conservation of cysteine residues ensures thiolate cluster formation, metal binding and provides both the structural and functional basis to identify these genes as MTs, cloning methods such as hybridization or amplification using degenerate oligonucleotides were ineffective because of its lack of similarity with other known crustacean MT sequences (Figure 6). The present study is the first to provide genomic DNA sequence information for a non-malacostracan crustacean, the first to report the promoter region of any crustacean MT, and one of the few to identify the metal-responsive element (MRE) consensus core sequence in any invertebrate. MREs were identified in the 5'flanking region of *D. pulex *MTs (Fig 6), Both Dpu Mtn1 and Dpu Mtn3 contained MREs within 200 bp of the transcriptional start site. The promoter region of the sea urchin MT gene was found to contain two MREs residing within 300 bp of the start of transcription [79,80]. There was only genomic (primary) sequence provided for one crustacean MT (i.e., green shore crab, *Carcinus maenas*) in the entire NCBI repository, but it lacked any flanking sequences (i.e., upstream and downstream regulatory regions). Green shore crab MT has a structure common to Dpu Mtn1 and Dpu Mtn2 with three exons separated by two introns [81]. In fact, similar architectures have been reported for the urchin, trout and several mammals. Also, *D. pulex *have much smaller intronic regions (Figure 6), likely a consequence of its compact genome size (~200 Mb). Although the architectures of these genes were similar to others, little sequence similarity was observed within their coding sequence for any known MT.

The translated daphniid sequences (i.e. *D. pulex*; *D. magna*, Poynton *et al.*[19]) also showed little similarity to other MT genes (Table 4, Fig 7), including other crustaceans. This includes the N-terminal crustacean motif (P- [GD]-P-C-C-x(3 or 4)-C-X-C;[41,48]. However, since daphniid MTs represent the first MTs isolated from a non-malacostracan crustacean, the lack of phylogenetic representation in the NCBI repository could account for some these differences. There was considerable similarity in terms of number and distribution of cysteine residues. All crustacean MTs sequenced to date are comprised of 18 cysteine residues with the exceptions of the copper MT isolated from the green crab [41], *D. magna *MT [19] and Dpu Mtn3 identified in this investigation that each contain 19 cysteines. The conservation of cysteine residues is expected, given that they are responsible for a great deal of the tertiary structure and metal-binding functions of the protein [78]. In fact, Valls *et al.*[41] has suggested using the binding properties (e.g., stoichiometry of metal- MT species) to strengthen current classifications that are based on phylogeny.

The current classification procedure for MTs [82] is based on phylogenetic relationships rather than amino acid composition and cysteine content. This nomenclature protocol was established to better identify related functional properties in which MTs are divided into families based on evolutionary conservation. In this classification scheme, crustaceans constitute a single family, given the name number three, which is comprised of three sub-families (crustacean one, c1; crustacean two, c2; crustacean, c), where c1 and c2 genes each constitute monophyletic clades. The third family termed, c, is reserved for crustacean MTs that are different from these others. *Daphnia pulex *MTs do not align well with malacostracan type 1 or type 2 MTs and thus, according to this nomenclature are designated Dpu Mtn1, 3, c; Dpu Mtn2, 3, c; and Dpu Mtn3, 3, c.

## Conclusion

In summary, the development of *D. pulex *cDNA microarrays and associated sequence information has provided a useful first generation functional genomic tool for examining biological responses of this key sentinel species to environmental agents and other stressors. Treatment of *D. pulex *with sub-toxic levels of cadmium revealed a specific pattern of gene expression changes that provide new insights into their biological and toxicological responses to this environmental contaminant. Moreover, microarray responses to cadmium led to the discovery of *D. pulex *MTs, whose gene structure and cysteine content clearly place it in this gene family, but whose sequence divergence reveals that classical cloning and sequencing techniques based on similarity were likely to fail. Further identification of *D. pulex *genes that are responsive to various experimental treatments through use of these genomics tools will provide new and important insights into their biology. Advances in *Daphnia *genomics will enable the further development of this species as a model organism for a wide variety of biological investigations.

## Methods

### Animals and cadmium exposure

*Daphnia pulex *(subclade arenata) used in this study were obtained from isoclonal laboratory cultures of an isolate collected from an ephemeral pond near the Pacific coast in Oregon. This pond is found north of Florence on the east side of highway 101 at milepost 201 in Douglas County. *Daphnia pulex *subclade arenata is a member of the *Daphnia pulex *complex [83] and our isolate is from the same population as the strain whose genome has been sequenced by the Joint Genome Institute as part of the *Daphnia *Genomics Consortium initiative [84]. The *Daphnia *were housed in 3L borosilicate glass beakers (20 per beaker) held inside an environmental chamber at a constant temperature (20 ± 1°C; [85]) and photoperiod (16:8 light-dark). Organisms were maintained in nanopure water reconstituted to moderate hardness [86] and renewed weekly. They were fed daily *Ankistrodesmus falcatus *at a rate of 75,000 cells/mL. Our pre-experimental procedure, described in Folt *et al.*[87], controlled for maternal effects in acute toxicity tests, demographic experiments and batch exposures. For these experiments, neonates (< 24 h old) were isolated from maintenance cultures one generation prior to metal exposure. These organisms are referred to as 'brood females', which were synchronized with respect to time of maturity for producing neonates for metal experiments.

#### Acute toxicity tests

Acute (48-h) toxicity tests were conducted with cadmium according to recommendations given by the United States Environmental Protection Agency with slight modifications [10]. Test solutions were prepared immediately prior to use with culture media from stocks made with CdCl_2 _(analytical grade, Sigma Chemical, St. Louis, MO, USA) dissolved in deionized water. Test concentrations nominally ranged from 1 to 150 μg Cd/L. Toxicity tests employed a completely random design consisting of five or six metal treatments and a control group arrayed in two-fold serial dilutions. Ten neonates (< 24 h old) were randomly placed into a 40 ml glass exposure chamber containing 30 ml of test solution. Four replicate exposure chambers were employed per treatment or control group. Daphniids were not fed during tests. Mortality was assessed for individuals in each container after 48-h exposure. An individual was labeled dead if it was unresponsive to gentle prodding with a pipette tip. These tests were repeated and results combined to determine lethal concentrations (LC *x *values, where *x *equals a given percent mortality) estimated from the probit transformed concentration-response curves.

#### Demographic experiments

Twenty-one day life-table experiments followed a completely randomized design as given in U.S. EPA [11]. A single organism was randomly placed into a 120 ml exposure chamber. Ten replicate chambers were used per test group. Treatment groups consisted of 0.25, 0.5, 1, 1.75, 2.5 μg Cd/L and controls. Test waters were renewed every other day, at which point general water quality parameters of temperature, dissolved oxygen, conductivity, and pH were monitored. Water column metal concentrations were measured at the beginning and end of each test. Mortality and reproduction were observed over the duration of the test and endpoints included age to first reproduction, cumulative reproduction, neonates per adult, and percent survival as per Chen and Folt [88]. Length measurements [89] and lipid ovary indices [57] were taken at the end of the experiment using an Olympus BX40 microscope fitted with a digital camera (Hitachi KP-D50) driven by Scion Image software (V. 1.63).

#### Batch exposure

The *Daphnia *used for microarray experiments and for validation tests by RT-PCR were exposed in repeated experiments in batches of 50 adult organisms plus their offspring per 3.5-l exposure chamber. The offspring of these animals were not discarded to increase biological variability and increase opportunities for gene discovery. Batch number was optimized to provide adequate sample mass for molecular evaluation (e.g., 1 adult *Daphnia *equals 1 μg of total RNA). Animals were introduced as neonates (< 24-h old) and cultures were maintained until they produced their third clutch (15-d). These experiments, which provided source material for microarray experiments and validation tests, included short-term (48-h) independently replicated (n = 3) exposures to non-lethal concentrations of cadmium (LC01, 20 μg Cd/L) and control conditions.

### Gene response profiles

#### Microarray construction

Working in the absence of abundant a priori DNA sequence data for *D. pulex*, we constructed a 3,842 element microarrays using 3,602 PCR-amplified cDNA and 240 control probes. A detailed description of this microarray platform is archived at the National Center for Biotechnology Information (NCBI) Gene Expression Omnibus under the accession number [GEO:GPL6195], series [GEO:GSE9746]. Briefly, cDNA for seeding the amplifications were obtained by directly transferring into the reactions 5 μl of cDNA bacterial transformants, which were grown in 1.2 ml of 2X YT and 0.005% chloramphenicol in 96-deepwell plates for 24 hours at 37°C. The arrayed cDNA clones were randomly picked from two high-quality *D. pulex *cDNA libraries (Creator SMART, Clontech). For details about the libraries and results from quality assurance tests, see Colbourne *et al.*[67]. The amplifications were conducted in 100 μl reactions containing 1× Taq buffer (Eppendorf), 0.2 mM dNTPs, 0.2 μM primers (Fwd. 5'-GTGTAAAACGACGGCCAGTAG 3' and Rev. 5'-AAACAGCTATGACCATGTTCAC 3'), 5 U Taq (Eppendorf). The PCR cycling conditions involved a 3 minute initial denaturation step at 94°C followed by 35 cycles of 94°C, 54°C and 72°C each for 1 minute. The products were purified using the Multiscreen-PCR 96-well system (Millipore) on a Biomek FX liquid handling robot (Beckman). Our pilot experiments for this section of our workflow indicated that this purification system recovered 80–90% of the original sample and introduced no impurities that interfered with immobilizing DNA onto glass. The quality of PCR amplifications was visually inspected by agarose-gel electrophoresis and their number and size were recorded using Kodak's 440cf scanner and 1D imaging software (v.3.6). The sample concentrations were determined by 96-well microplate spectrophotometer (Molecular Devices, SpectraMax 190) and adjusted to 50–200 ng/μl for printing. The PCR amplifications were classified as having produced a single high yield product (3,238; 91%), as failures (163 reactions; 5%), as having produced more than one amplicon (102 reactions; 3%) or as being weak (49 reactions; 1%). The DNA yields averaged 409.7 ng with a standard deviation of 210.8 ng. Printing was achieved using an Omnigrid 100 robot (GeneMachines). The cDNA were spotted in tandem on GapsII amino-silane slides (Corning) in 3× SSC and 1.5 M Betaine buffer using Stealth Micro-Spotting Pins (Telechem) at 20°C and 65% humidity. The cDNA was fixed to the microarray slides by baking at 85°C for 3 hours. To achieve minimal signal to background ratios averaging 40–50 fluorescence units, the slides were post-processed by washing in 5 × SSC buffer with 0.1% SDS at 55°C for 5 minutes, rinsing in water at room temperature for 2 minutes, denaturing the DNA in water at 95°C for 4 minutes, then rinsing in water at room temperature for 30 seconds. The slides were finally rinsed in isopropanol at 4°C and dried by centrifugation at 500 g for 5 minutes before being stored. Slides were printed in groups of 100 or 120, where 95% of the slides were free of defects. Negative controls were included, designed to detect potential problems. To test for the cross-contamination of probes, printing buffer containing no DNA was first deposited at the beginning of each subarray. Printing buffer was also printed following positive control DNA (coding cytochrome c, cytochrome b, actin and ferritin) at both the beginning and end of the subarrays. To test for the effect of template DNA during the hybridizations, the product of intentionally failed PCR reactions with template DNA but no primers were printed. Finally, amplified DNA from *Arabidopsis *and lambda phage plus bacterial spiking controls (Ambion) was also included. Based on random sequencing of 619 cDNAs probes, and post hoc sequencing of an additional 927 cDNA probes, gene-redundancy on the array was calculated to be roughly 57%. Thus, there were likely ~1,550 interrogated unique genes on the array.

#### Labeling and hybridization

Microarrays were used to discover genes that were differentially expressed following sub-lethal cadmium stress in *D. pulex*. They utilized RNA isolated from three independent and concurrently replicated exposures of *Daphnia *to cadmium and control conditions, applied to three replicate microarrays using a standard control vs. treated design that included dye swaps. This design provided three replicates of metal exposed and control *D. pulex*, with the technical variability of hybridization kinetics captured on each slide across duplicate probes.

Total RNA was isolated from *Daphnia *that were acutely (48-h) exposed to 20 μg Cd/L (treated) and from their genetic clones reared under standard (control) conditions as given above for batch cultures. Animals were directly placed in lysis buffer and RNA was extracted using Qiashredder columns and RNeasy kits (Qiagen). DNA contamination was removed by DNAse treatment (Ambion) and RNA was quantified by spectrophotometry (Nanodrop Technologies); and quality determined with a Bioanalyzer 2100 (Agilent). For each sample, 10 μg of total RNA was reverse transcribed with random hexamer primers using SuperScript II (Invitrogen) and an overnight incubation, which included aminoallyl-dNTPs. Following reverse transcription and clean up (alkaline hydrolysis and Qiaquick columns, Qiagen), cDNA samples were coupled to Alexa Fluor dyes (555, 647), using amino-allyl labeling methods and alternating direction with replicate arrays (i.e., dye-swap; [90]). The amounts of dye incorporated cDNA were measured by spectrophotometer using the acceptability cutoffs of > 200 pmol of dye incorporation and a nucleotide to dye molecule ratio of > 50 [91]. The labeled samples were then pooled according to treatment comparisons (e.g., control vs. Cd treated), dried, and resuspended in hybridization buffer (50% formamide, 5× SSC, 0.1% SDS, 20 μg SSDNA, and 20 μg of poly(A)-DNA). The hybridization solution (containing the dye labeled samples was placed on the microarray and hybridization was accomplished overnight at 42°C in a specially fitted hybridization chamber (Corning). Following hybridization, the glass slides were washed successively in a low stringency solution (1× SSC, 0.2%SDS) at 42°C for four minutes, high stringency solution (0.1× SSC, 0.2%SDS) at room temperature for four minutes, twice in 0.1× SSC for 2.5 minutes, and finally they were dipped in water, dried and stored in the dark until fluorescence was measured [91].

#### Gene Expression Analysis

The fluorescent signals were scanned and the array data were extracted using GeneChip Scanner 3000 software version 5.1 (Axon). The data were coupled to the array template using the GeneChip Operating System (GCOS) software, V. 1.4. For each microarray, LIMMA functions as defined in [92-94] were used to create unique, background subtracted, LOESS (i.e., locally weighted least squares regression) normalized, log expression values, which accounted for duplicate spots using gene-wise linear models fit to expression data. The LIMMA derived log expression values were fit to a linear model based on treatment, which moderated the resulting t statistics using the empirical Bayes method. Probes with a p-value ≤ 0.05 were deemed significant and selected for sequencing. A 5' expressed sequence tag was generated for the majority of the differentially expressed clones. These sequences were analyzed and assigned putative gene function annotations based on sequence similarity searches against NCBI protein and insect genome databases [67]. Analyses of the functional grouping of genes based on Gene Ontology (GO) assignments were performed using Blast2GO [44]. For those probes sharing common putative annotations, we performed permutation tests to estimate the likelihood that random processes would place these highly represented annotations on our list of differentially expressed cDNA and to establish p-values on certain annotations (i.e., P values provided in Table 3).

#### Confirmation of genes regulated in response to cadmium exposure

Quantitative real-time (RT) PCR was used to validate expression levels of four genes identified on the microarray; three genes were regulated following cadmium exposure (i.e., cuticle protein-2, Contig 257; 2-domain haemoglobin protein subunit, Contig 262; metallothionein, Contig 221) and one gene for which expression was not altered (i.e., serine-threonine kinase, Contig 274). This validation test included a set of replicate RNA from the microarrays plus six independent biological replicates. Primers and *TaqMan *probes were designed using PRIMER EXPRESS, V (Applied Biosciences), which are listed in Table 3. Reverse transcription was performed with the Omniscript reverse transcription kit from Qiagen. Two micrograms of total RNA was reverse transcribed for each sample using random primers (final concentration of 5 μM) for metallothionein and gene-specific primers for the other three genes (0.5 μM final concentration, Table 3). Real-time PCR was performed following the Qiagen protocols on the Applied Biosystems 7700 machine and included a standard curve in each run for each gene amplified in that run. The standard curve consisted of serial dilutions of the cDNA being amplified. Applied Biosystems Master Mix was used in each amplification, which contained all PCR components necessary except the cDNA, primers (900 nM each, final concentration) and the Taqman probe (250 nM, final concentration). Taqman probes were FAM labeled and contained an MGB quencher. Controls to test for DNA contamination were always included, even though DNase digestion was performed on the RNA before reverse transcription. The amplification steps consisted of the standard 40 cycles preceded by two minutes at 50°C and ten minutes at 95°C to activate the enzyme. Each cycle included 15 seconds at 95°C and 1 minute at 60°C. For each sample, the cycle at which amplification reached the exponential phase was recorded as the Ct value. Final level of transcript for metallothionein was normalized using the Nanodrop spectrophotometer to directly measure the cDNA level in each sample. The other genes were normalized to the levels of serine threonine kinase, which was not differentially regulated.

### Metallothionein characterization

#### cDNA and genomic sequence determination

Quality EST sequences were obtained from 1,529 cDNA elements on the array. These were clustered, their open reading frames were determined and they were annotated based on sequence similarity searches against NCBI and custom protein databases. Details on our methods and of the results are presented elsewhere [67]. The sequences of two elements on the microarray that respond to cadmium stress revealed identical transcripts for a cysteine-rich protein resembling metallothionein. The translated gene sequence was used to identify additional metallothionein-like loci within a sequence database derived from 36 cDNA libraries used to support the ongoing *D. pulex *genome sequencing project (Colbourne et al., in prep). The program tBlastn [95] was used at a significance cut-off value of e < 1 × 10^-10 ^to search 36,342 sequence assemblies (EST clusters). To determine whether the identified sequences were homologous or alternatively spliced loci, these were subsequently matched to the latest *D. pulex *genome sequence assembly by the Joint Genome Institute using the Blastn tool on wFleaBase [96]. The cDNA was aligned to genomic DNA sequences by the Clustal method using MegAlign (DNASTAR, Inc.).

#### Phylogenetic analysis

Metallothionein protein sequences for 18 selected insect and crustacean species were obtained from the NCBI database. The multiple cDNA alignment was produced by the MAFFT version 5 program using the E-INS-i strategy [97]. The scoring matrix for the protein sequences was Blosum62 and the alignment parameters included a gap opening penalty of 3 and a gap extension penalty (offset value) of 0.15. This protein alignment was then used to calculate a genetic similarity matrix including all loci by using MEGA version 3.1 [98] with a Poisson correction of the distances and pairwise deletion of the alignment gaps. A corresponding neighbor-joining tree was constructed that included 1,000 bootstrap pseudo-replicates of the data for assigning confidence to nodes. A maximum likelihood phylogeny was also constructed by quartet puzzling using the program Tree-Puzzle version 5.2 [99] with 10,000 puzzling steps and with the Dayhoff model of amino acid substitution under uniform rates of molecular evolution.

## Authors' contributions

JRS, JKC, JCD, CYC, CLF and JWH conceived the study and designed the experiments and subsequent analyses. JRS and SPG performed cadmium exposures and toxicity assays, JRS and JCD conducted the microarray experiments and THH performed the statistical analyses. JRS drafted the manuscript and all authors contributed to, improved upon, and read and approved the final version.
